# Carbamylated Proteins in Renal Disease: Aggravating Factors or Just Biomarkers?

**DOI:** 10.3390/ijms23010574

**Published:** 2022-01-05

**Authors:** Laëtitia Gorisse, Stéphane Jaisson, Christine Piétrement, Philippe Gillery

**Affiliations:** 1 MEDyC Unit CNRS UMR n° 7369, Faculty of Medicine, University of Reims Champagne-Ardenne, 51092 Reims, France; lagorisse@gmail.com (L.G.); sjaisson@chu-reims.fr (S.J.); cpietrement@chu-reims.fr (C.P.); 2 Biochemistry Department, University Hospital of Reims, 51092 Reims, France; 3 Pediatrics Department, University Hospital of Reims, 51092 Reims, France

**Keywords:** carbamylation, renal diseases, pathophysiology, complications, biomarkers

## Abstract

Carbamylation is a nonenzymatic post-translational modification resulting from the reaction between cyanate, a urea by-product, and proteins. In vivo and in vitro studies have demonstrated that carbamylation modifies protein structures and functions, triggering unfavourable molecular and cellular responses. An enhanced formation of carbamylation-derived products (CDPs) is observed in pathological contexts, especially during chronic kidney disease (CKD), because of increased blood urea. Significantly, studies have reported a positive correlation between serum CDPs and the evolutive state of renal failure. Further, serum concentrations of carbamylated proteins are characterized as strong predictors of mortality in end-stage renal disease patients. Over time, it is likely that these modified compounds become aggravating factors and promote long-term complications, including cardiovascular disorders and inflammation or immune system dysfunctions. These poor clinical outcomes have led researchers to consider strategies to prevent or slow down CDP formation. Even if growing evidence suggests the involvement of carbamylation in the pathophysiology of CKD, the real relevance of carbamylation is still unclear: is it a causal phenomenon, a metabolic consequence or just a biological feature? In this review, we discuss how carbamylation, a consequence of renal function decline, may become a causal phenomenon of kidney disease progression and how CDPs may be used as biomarkers.

## 1. Introduction

Renal disease is a major public health burden, affecting millions of people worldwide. The Global Burden of Disease study showed that 1.2 million people died of chronic kidney disease (CKD) in 2017, with a 41.5% rise between 1990 and 2017 [1], and acute kidney injury (AKI) is thought to contribute to around 1.7 million deaths every year [2]. Diabetes and hypertension are the leading causes of glomerular lesions and advanced kidney disease in all high-income and middle-income countries [3]. The progressive decline in kidney function is related to the occurrence of pathophysiological mechanisms that include glomerulosclerosis, tubular atrophy and interstitial fibrosis.

As kidney disease progresses, residues of organic compounds, named “uremic toxins”, accumulate in the bloodstream and exert adverse biological effects. The retention of such compounds leads to uremic syndrome, which is characterized by the loss of biological functions, contributing to disease progression. A wide number of uremic toxins have been identified in CKD patients, among which is urea [4,5,6]. While direct urea toxicity has been controversial—described as well tolerated or, conversely, harmful [7]—it is established that urea promotes indirect adverse effects through carbamylation [8].

Carbamylation is an irreversible nonenzymatic post-translational modification (NEPTM) occurring between isocyanic acid and the amino groups found on lysine residues or the N-terminal extremity of proteins, peptides or free amino acids [9]. Isocyanic acid mainly derives from the spontaneous dissociation of urea into ammonia and cyanate in aqueous solutions. This reversible dissociation is in favour of urea, with the equilibrated ratio being about 1:100. Cyanate is converted into its tautomer isocyanic acid, which is highly reactive [10]. In fact, isocyanic acid immediately binds to proteins and consequently moves the equilibrium toward dissociation. The chronic elevation of urea encountered in CKD patients leads to an amplified cyanate generation and therefore protein carbamylation. Isocyanic acid may also be formed from thiocyanate under the action of myeloperoxidase (MPO) in the presence of hydrogen peroxide, both of which are released by leukocytes. This pathway promotes carbamylation preferentially in inflammatory sites, especially in atherosclerotic plaques [11]. Minor environmental sources have been described. Cyanate intake might result from breathing the air contaminated by tobacco smoke and biomass combustion [12] or from the consumption of food containing cyanogens, which are metabolized to cyanate [13].

Even if protein carbamylation is primarily influenced by urea concentration, it depends on other endogenous features, such as exposure time to hyperuremia [14], protein lifespan and turnover [15,16], and competition with other NEPTMs, such as glycation [17,18]. Glycation corresponds to the binding of sugars (or their by-products) to amino groups followed by molecular rearrangements, resulting in the formation of advanced glycation end-products (AGEs). Carbamylation and glycation actually target the same protein residues and may concurrently occur. In vivo experiments demonstrated that these two irreversible modifications compete with each other on circulating proteins [17] as well as on tissue proteins [18] in CKD diabetic mice. Nevertheless, carbamylation appeared to take precedence over glycation, suggesting the presence of complex interactions in the development of long-term complications of CKD and diabetes.

Numerous studies have provided evidence of the structural and functional alterations of proteins, as well as of inappropriate cell responses, caused by carbamylation [19]. Furthermore, carbamylated-derived products (CDPs) have been identified at high concentrations in patients with CKD [19,20,21]. However, the mechanisms involved in carbamylation-induced urea toxicity in renal patients lack clarity, unlike other uremic toxins. Indeed, AGEs and hippuric acid are involved in cardiovascular complications, whereas indoxyl sulfate and p-cresyl sulfate are responsible for inflammation and fibrosis in renal tubular cells [5,6,22]. Thus, the cause–consequence link between these uremic toxins and kidney disease progression is well established. However, the comparable relevance of the carbamylation reaction is still unclear. Is carbamylation a causal phenomenon, a metabolic consequence or just a biological feature? Even if this modification is more likely to be considered a metabolic consequence of kidney function decline and a generator of biomarkers for renal deficiency, much evidence suggests its participation in disease pathophysiology. In this review, we describe carbamylation as a physiological process amplified during CKD, we emphasize the causal role of carbamylated proteins in CKD progression and we review the use of CDPs as biomarkers of poor clinical outcomes.

## 2. Carbamylation, a Physiological Process Increased in Chronic Kidney Disease

### 2.1. Carbamylation Is a Physiological Process

Urea is a waste molecule that facilitates nitrogen excretion in humans. It is generated primarily in the liver from ammonia, a product of protein catabolism. Urea diffuses freely through cell membranes and its concentration in cellular fluids is equal to that in plasma [23]. Thus, carbamylation is an unavoidable reaction affecting intra- and extracellular proteins. Indeed, CDPs were found in different cell types, such as renal inner medulla cells, fibroblasts and vascular smooth muscle cells (VSMCs) [16,24,25]. Cell immunostaining has highlighted carbamylated proteins throughout the cells [24] and mass spectrometry has helped to identify them in several subcellular compartments, including the centrosome, nucleus, Golgi apparatus, endoplasmic reticulum, mitochondria and plasma membrane [25,26]. Circulating proteins (haemoglobin, albumin and lipoproteins [20,27,28]), as well as extracellular matrix (ECM) proteins [16,29], were also found to be modified by carbamylation. However, some long-lived proteins, such as type I collagen and elastin, are longer exposed to isocyanic acid and are consequently more prone to carbamylation. Given that this NEPTM is irreversible, carbamylated matrix proteins accumulate in the organism, particularly in ECM-rich tissues, such as skin and the aorta. In vivo studies showed a higher and faster accumulation of CDPs in skin with age in humans, bovine and mice, regardless of lifespan [16]. CDPs were found in several other tissues in rodents (i.e., brain, heart, aorta, skeletal muscle, liver, bone and kidney [15,29]) and humans (i.e., the aorta [16]). Hence, carbamylation has a physiological occurrence and is a ubiquitous ageing process, similar to other NEPTMs, such as glycation and oxidation [30,31].

The MPO pathway, though minor compared to the urea pathway, might take part in the carbamylation of circulating proteins during ageing. Serum concentrations of MPO were shown to increase with age, along with the increase in oxidative stress [32,33]. Interestingly, serum carbamylated proteins were positively correlated with malondialdehyde, a marker of imbalanced oxidative stress, in a cohort of young and old healthy humans [34]. Carbamylation appears to be closely associated with age-related oxidative stress, likely via MPO activity. In addition, some authors have proposed considering serum CDPs as markers of biological age, since they were correlated with an aging functional immunological signature [34].

### 2.2. Chronic Kidney Disease Exacerbates Carbamylation

The progressive kidney fibrosis and nephron mass reduction encountered in CKD leads to a decline of kidney function and therefore to increased urea concentrations. Consequently, isocyanic acid formation is enhanced. While the concentration of isocyanic acid in healthy subjects is about 45 nmol/L, it reaches 140 nmol/L in uremic patients and decreases two-fold after haemodialysis session [35]. These significant increases in isocyanic acid amounts greatly favour the carbamylation of proteins, enhancing the interest of nephrology researchers in this reaction. In the early 1990s, in vivo investigations identified carbamylated haemoglobin in patients with end-stage renal disease (ESRD) [36]. Since then, the carbamylation of other circulating proteins, such as albumin, low-density lipoproteins (LDLs) and high-density lipoproteins (HDLs), was evidenced in patients with CKD [20,28]. Tissue protein carbamylation was described as well. In the first place, immunohistochemical analyses revealed the presence of CDPs in the renal biopsies of patients, especially in glomerular and tubular cells [37]. Later, in vivo studies carried out in our laboratory demonstrated the widespread accumulation of CDPs during experimental CKD [15]. Indeed, carbamylated protein content remained significantly higher in a set of tissues 20 weeks following subtotal nephrectomy compared to control mice. As observed in healthy old animals, tissues containing a large proportion of long-lived ECM proteins depicted the most important accumulation of CDPs.

Beyond the urea pathway, carbamylation can occur through an enhancement of the non-uremic MPO process during renal disease. Plasma concentrations of thiocyanate were found to be six times greater in CKD patients, even after a haemodialysis session [38]. Therefore, the oxidation of thiocyanate by MPO promotes protein carbamylation preferentially at sites of inflammation and atherosclerotic plaques, since MPO is released from neutrophils, monocytes and macrophages [11].

## 3. Carbamylation and Renal Disease Progression: The Consequence Becomes the Cause

**Figure 1:**
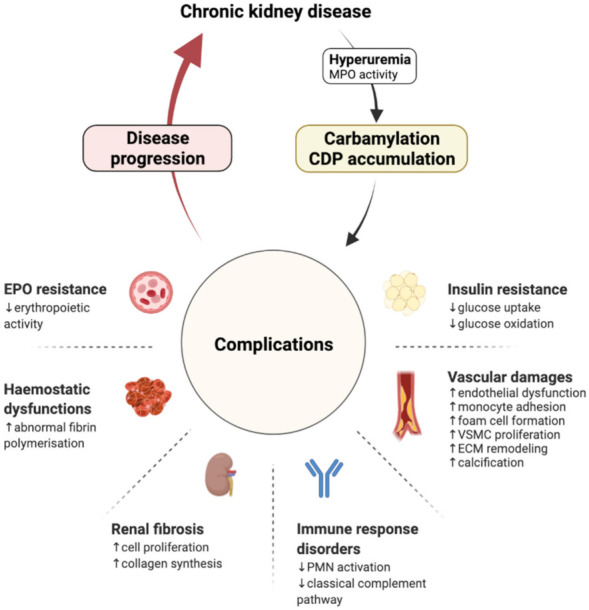
Carbamylation, a consequence of renal function loss and an aggravating factor of CKD progression. CDP: carbamylation-derived product; ECM: extracellular matrix; EPO: erythropoietin; MPO: myeloperoxidase; PMN: polymorphonuclear neutrophil; VSMC: vascular smooth muscle cell.

Numerous studies have revealed the mechanisms by which carbamylated proteins could cause CKD complications and progression (Figure 1, Table 1).

### 3.1. Cardiovascular Damages

Cardiovascular diseases (CVDs) are common complications in patients with ESRD and are a major cause of morbidity and mortality [3]. CKD is associated with several traditional (such as hypertension and diabetes mellitus) and non-traditional (such as endothelial dysfunction and inflammation) risk factors for CVDs, which accelerate the progression of the disease [63]. Evidence has emerged demonstrating the link between carbamylation and the major pathophysiological mechanisms associated with CVDs (i.e., lipoprotein metabolism, cellular effects, ECM remodelling and calcification).

#### 3.1.1. Lipoprotein Metabolism

Several clinical studies have evidenced high plasma concentrations of carbamylated LDLs (cLDLs) and carbamylated HDL (cHDLs) in patients receiving haemodialysis treatment [28,40,45]. In vitro, it was shown that apolipoprotein B carbamylation decreased LDL affinity for its apoB/E receptor [64] and delayed LDL clearance [39]. This impaired metabolism promoted a redistribution of cLDLs in organs, particularly in the vascular system [43]. On the other hand, the presence of carbamylated proteins was evidenced within atherosclerotic plaques suggesting a correlation between carbamylated proteins and atherosclerosis [11]. Since then, several studies have demonstrated the pro-atherogenic role of carbamylated lipoproteins, namely by promoting endothelial dysfunction, monocyte adhesion, foam-cell formation and VSMC proliferation, as discussed in the following paragraph [11,26,29,40,41,42,44,45,49,50].

#### 3.1.2. Cellular Effects

Endothelial cells, monocytes/macrophages and VSMCs are major cellular contributors to the pathogenesis of atherosclerosis [65] and are all impacted by carbamylated lipoproteins.

Atherosclerotic lesions are initiated by a dysfunctional vascular endothelium, a process accelerated by cLDLs, which induce oxidative stress and the cell death of endothelial progenitor cells [66] and aortic endothelial cells [40,41]. Similarly, cHDLs contribute to endothelial injury by reducing endothelial cell proliferation, migration and angiogenesis ability [45]. Carbamylation not only deprives the original protective function of HDLs, but also converts them to proatherogenic particles.

Another early cellular event in atherogenesis is the focal recruitment of circulating monocytes. cLDLs favour monocyte adhesion through their binding to LOX-1 on endothelial cells, leading to the subsequent overexpression of ICAM-1 and VCAM-1 [42,43]. cLDLs have also a high affinity for the scavenger receptors CD36, SREC-1 and SR-A1, promoting the translocation of cLDLs beneath the aortic endothelium [43]. Once in the subendothelial area, cLDLs stimulate the lipid loading of macrophages and foam cell formation via SR-A1 [11]. cHDLs promote the formation of foam cells, as well, by impairing cholesterol efflux [46,47].

Finally, cLDLs participate in the disease progression by increasing VSMC proliferation and upregulating ICAM-1 and VCAM-1 expressions [40,44]. These cell adhesion molecules are involved in inflammatory reactions since they induce leukocyte accumulation and mononuclear cell activation [67].

Overall, by promoting a proatherogenic environment (with disturbed endothelium integrity, foam cell formation and inflammation), carbamylated lipoproteins appear to be highly involved in the accelerated CVD progression in CKD. Notwithstanding the significance of these data, it is important to mention that most of the in vivo and in vitro studies were performed using lipoproteins carbamylated by incubation, with high KCNO concentrations ranging from 1 to 10 mM. Even if this carbamylation process might not mimic physiological conditions, it allows for the comprehension of cellular mechanisms and the adverse outcomes observed in patients.

#### 3.1.3. ECM Remodelling

The carbamylation of ECM proteins participates in the alteration of vascular ECM homeostasis, a major event in cardiovascular complications [68]. The enrichment of arterial walls in collagen fibres, especially type I fibres, is a critical characteristic of atherosclerosis progression. In vivo studies performed in our laboratory have demonstrated that type I collagen was a preferential substrate for carbamylation because of its long half-life and was highly carbamylated during hyperuremia [15,16]. One of the consequences of type I collagen carbamylation is the enhancement of monocyte adhesion and matrix metalloproteinase (MMP)-9 production and activation [48]. The secretion of proteolytic enzymes, such as MMP-9, by macrophages is known to increase elastin degradation and plaque disruption [69]. Thus, carbamylated type I collagen could indirectly participate in plaque rupture. However, it could directly take part in plaque erosion. Biophysical studies have revealed local conformational changes in the triple helix of in vitro carbamylated collagen with dramatic consequences for fibrillogenesis and fibre network formation [49]. Important to note here is that the carbamylation rate of collagen was suggested to be similar to that reported for the carbamylated serum proteins of uremic patients [53,70]. Given that the mechanical properties of collagen are highly disturbed, carbamylation may confer a rupture-prone status to the plaques. Interestingly, aortic elastin was also shown to be carbamylated in vivo in a murine model of diet-induced carbamylation. The presence of CDPs in the aorta was concomitant with an increase in elastin lamellae stiffness, and aortic pulse wave velocity was increased in apoE knocked-out mice receiving the same diet [29]. Mechanical and functional properties of carbamylated elastin, such as sensitivity to MMPs, are still to be determined.

#### 3.1.4. Calcification

Recent studies have brought new insights into the involvement of carbamylation in the development of vascular calcifications. In vitro studies have showed that global protein carbamylation reduced the mitochondrial membrane potential and exacerbated mitochondria-derived oxidative stress in VSMCs. As a result, ENPP1 was downregulated, PPi generation was reduced and the inhibition of mineralization abolished. In vivo investigations confirmed the presence of mitochondrial dysfunctions and aortic calcifications in a carbamylated rat model [26]. Two other studies identified specific targets for carbamylation that participate in vascular calcifications. The ubiquitous sortilin, a trafficking and cell signalling protein, was found to be carbamylated in the calcified arteries in patients with CKD [51]. Mechanistically, studies using coronary artery SMCs revealed that the carbamylation of the soluble form of sortilin increased ALPL and RUNX2 expressions, augmented tissue non-specific alkaline phosphatase (TNAP) activity and therefore promoted matrix calcification. A clinical approach confirmed the association between carbamylated soluble sortilin and coronary artery calcification volume progression after a 4-year follow-up in patients with kidney function decline [51]. The carbamylation of uromodulin was discovered as well [50]. The study demonstrated, in vitro and in vivo, that carbamylation hindered the osteogenic interference function of uromodulin. Indeed, carbamylated uromodulin failed to bind and trap pro-inflammatory cytokines, TNFα and IL-1β, which trigger a signalling pathway, allowing the osteo-/chondrogenic transdifferentiation of VSMCs [50].

### 3.2. Renal Fibrosis

Proteinuria is associated with nephrotoxicity, including, specifically, albuminuria, which was reported to contribute to tubulointerstitial damages [71]. The carbamylation of proteins was shown to amplify these proinflammatory and profibrotic mechanisms and therefore the severity of renal damage. In the axolotl amphibian model, in vitro carbamylated albumin caused intense tubuloepithelial and interstitial lesions, mediated by the upregulation of profibrotic factors (e.g., PDGF, TGF-β, NF-κB, EGF, and ET-1) [52]. In addition, carbamylated serum proteins increased rat mesangial cell proliferation and the synthesis of type I and type IV collagens, at a carbamylation rate that was assessed to be similar to that of serum proteins in uremic patients [53]. Altogether, these data suggest that the carbamylation of proteins favours interstitial fibrosis by triggering inappropriate renal cell responses. Furthermore, carbamylation could participate in renal fibrosis through direct actions on ECM. As previously mentioned, carbamylated type I collagen depicts structural changes, altering its mechanical properties and its turnover, favouring fibrosis [49]. Other collagens found in renal tissue might be prone to carbamylation and to conformational alterations, which could contribute to fibrosis. Significantly, the presence of CDPs was observed in rat kidneys (in the cortex, outer medulla and extensively in inner medulla) [25], and with a much higher intensity in the nephrectomised than in control mice [15].

### 3.3. Haemostasis Dysfunctions

The coagulation process and clot formation involve an integrated system that includes the vascular wall, the platelets, the coagulation system and the fibrinolytic system [72]. Haemostasis is commonly disturbed in CKD patients, resulting in thrombotic complications [73]. Carbamylation may contribute to haemostasis troubles by promoting endothelial dysfunction [40,41,66], but also through fibrinogen modifications. As with other circulating proteins, fibrinogen was found to be highly carbamylated in a cohort of patients under haemodialysis, and in vitro investigations reported functional consequences caused by carbamylation [55]. Although carbamylated fibrinogen remained able to be cleaved by thrombin into fibrin, the clot architecture was modified, the cross-linking process was impaired and fibrin polymerization slowed down. In addition, the thick, matted layered clot was more resistant to plasmin-induced lysis. These data suggest that the carbamylation of fibrinogen may participate in the uncontrolled expansion and instability of the clot, and finally to thrombotic events. Some authors have also suggested that carbamylated fibrinogen leads to inflammatory events, since carbamylated fibrinopeptide A, released from fibrinogen after thrombin cleavage, gained chemotactic activity for neutrophils and fibroblasts [55].

### 3.4. Immune Response Disorders

Immune system dysfunction is one of the more serious complications of CKD, and involves both adaptative and innate immunities, initiating and promoting the persistence of systemic inflammation [74]. Carbamylation was shown to disturb the innate system, notably by impacting polymorphonuclear neutrophils (PMNs) functions. In vitro studies have demonstrated that carbamylated type I collagen strongly inhibited focal adhesion kinase (FAK) phosphorylation, NADPH oxidase activation and reactive oxygen species (ROS) production [49]. Similar findings for PMN functions were obtained with carbamylated albumin [75]. Moreover, PMNs collected from uremic patients showed a reduced ability to generate superoxide in response to stimuli (*Staphylococcus aureus*, N-formyl peptide or phorbol ester) [76], which led to an enhanced risk of bacterial infections in ESRD patients [77]. Thus, PMN respiratory burst inhibition, caused by carbamylated collagen and albumin, could explain the high occurrence of infections and inflammatory syndromes in patients with CKD.

Aside from its influence on PMN metabolism, carbamylation disturbs the innate immunity by promoting complement pathway disorders. A study reported that the carbamylation of immunoglobulin G (IgG) Fc fragment by 100 mM KCNO hindered the ability of IgGs to bind C1q, the first component of the classical complement pathway, and thus to inhibit complement activation [60]. Improper complement activation leads to a decrease in immune complex clearance, an event observed in uremic patients [78]. Hence, it is relevant to speculate that the carbamylation of IgGs could have an impact on the susceptibility of CKD patients to infections. Importantly, the carbamylation of IgGs occurs in vivo, as demonstrated by mass spectrometry analyses, which have helped to identify carbamylated IgGs in the synovial fluid of rheumatoid arthritis patients [60].

Adaptative immunity is impaired by carbamylation as well. The structural modifications of proteins induced by carbamylation promotes the generation of neo-epitopes and the subsequent formation of anti-carbamylated protein antibodies (anti-CarP). This phenomenon has been well described in rheumatoid arthritis, where anti-CarPs constitute diagnostic and prognostic markers of the disease, with the presence of anti- CarP being predictive of a more severe clinical course [79]. Several carbamylated proteins have been described as targets for the production of anti-CarP, namely, albumin [80], filaggrin [81], collagen [82], vimentin [83,84], α-enolase [85], alpha2-macroglobuline and hemopexin [86].

### 3.5. Erythropoietin Resistance

CKD anaemia is largely derived from decreased erythropoietin (EPO) production due to the failure of kidney functions and iron deficiency [87]. Carbamylation is also behind functional alterations, and is characterized by a lack of EPO bioactivity through a defective binding to the EPO receptor homodimer or monomer on myeloid cells [56]. In vivo, a weekly injection of carbamylated EPO (cEPO) over 3 or 8 weeks did not increase the haematocrit levels of rats or mice [56,57]. In this study, the carbamylation of EPO was carried out with low cyanate concentrations (i.e., 1.5 µM); however, the carbamylation rate of cEPO was not compared with endogenous cEPO from uremic animals. Nevertheless, these data indicated that the carbamylation of EPO could contribute to anaemia by preventing erythropoiesis. In addition, a clinical study performed on a cohort of haemodialysis patients brought forward evidence on the contribution of carbamylation to EPO resistance. A high level of albumin carbamylation was associated with the erythropoietin resistance index (ERI) and predicted EPO resistance. Interestingly, carbamylated albumin was considered to be a stronger predictor of death than ERI [88].

Surprisingly, it was established that cEPO kept its tissue protective properties by interacting with a heterodimer receptor, EPOR/CD31 [89,90]. This effect was shown to be mediated by reduced ROS generation in endothelial cells [91], metabolic stress resistance in brain and heart cells [92], decreased cell apoptosis and an inhibited interstitial fibrosis in kidneys [93]. In addition, in a rat model of AKI, the injection of cEPO induced minimal apoptosis, but caused a proliferative rise in the number of tubular cells and an increase in peritubular capillary formation, yet without increasing haemoglobin concentrations [94,95]. Therefore, it is thought that the administration of cEPO could provide renoprotection in patients with nephropathy without the detrimental effects of high EPO doses, such as procoagulant and prothrombotic effects [96].

### 3.6. Insulin Resistance

Carbamylation was shown to participate in insulin resistance (IR), a well-known complication in CKD patients. Firstly, carbamylation was shown to directly impact insulin bioactivity by impairing its receptor-binding capacity in rat hepatocytes and glucose oxidation in rat adipocytes [58]. Secondly, carbamylation was indirectly related to IR because of carbamylated free asparagine (c-L-Asn). The preincubation of rat adipocytes with c-L-Asn interfered with insulin-sensitive glucose uptake. Specifically, c-L-Asn preincubation reduced the glucose transporter GLUT4 activity, with the underlying mechanism(s) being unresolved [59]. Notably, c-L-Asn compounds were found at high concentrations in the plasma of CKD patients and may contribute to IR present in uremic patients [59].

## 4. How Can CDP Formation and Accumulation Be Reduced?

**Figure 2:**
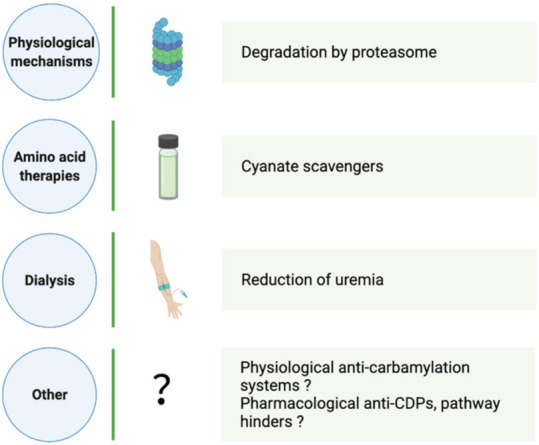
Mechanisms and strategies to limit the carbamylation process and to reduce CDP accumulation. CDP: carbamylation-derived product.

Given the harmful potential of NEPTMs, especially when they are amplified in the context of chronic diseases, physiological elimination processes and interference strategies have been widely investigated. Regarding the case of the well described glycation reaction, various anti-AGEs approaches (e.g., AGE crosslink breakers, AGE receptor antagonists) have demonstrated promising beneficial effects in animal models. Due to the causative role of AGEs and their relevance in pathophysiology, these preclinical studies have been translated into clinical trials in order to lower the burden of diabetes mellitus, diabetic nephropathy and metabolic syndrome [97]. Given the involvement of CDPs in disease progression, CKD, CVD and rheumatoid arthritis, the limitation of protein carbamylation is of great interest (Figure 2). Significantly, the risk of mortality associated with amplified carbamylation during CKD has induced a growing number of investigations into carbamylation prevention and elimination.

### 4.1. Physiological CDP Elimination

The organism is able to remove proteins irreversibly modified by other NEPTMs, such as glycation and oxidation, or to prevent these reactions for the optimal functioning of cells and tissues [98]. By contrast, CDP elimination processes are barely known. Nevertheless, a few investigations have shown that CDP elimination depends notably on the protein half-life and turnover. Hence, the carbamylated matrix, plasma or intracellular proteins are not removed at the same speed.

Using a mouse model, our laboratory demonstrated that skin type I collagen is extensively carbamylated in cyanate-fed mice. After a return to a standard diet for 9 weeks, carbamylated plasma protein concentrations were reduced by 99%, whereas skin type I collagen exhibited a 45% reduction in the carbamylation rate [16]. The processes by which carbamylated matrix proteins are discarded are not known and need further investigation. We can speculate about the contribution of some processes involved in the renewal and remodelling of ECM proteins.

At the cell level, intracellular CDPs are rapidly removed from fibroblasts through the proteasome pathway [24], and other control systems (e.g., chaperones and repair enzymes) are not excluded. The same processes likely occur in renal cells, where the presence of CDPs has been evidenced [25]. However, renal cells are exposed to high concentrations of urea-generating carbamylated proteins to a large extent. We can hypothesize that the systems responsible for the control of protein homeostasis become overwhelmed, creating ubiquitinated aggregates and inducing cell dysfunctions and cell death, a case that occurs during extensive oxidative stress [99].

### 4.2. Aid in Carbamylation Limitation

#### 4.2.1. Dialysis

In a large-scale study, patients on maintenance haemodialysis over one year had a marked reduction of carbamylated albumin (cAlb) concentrations, with a 52% decrease after 3 months [21]. By contrast, cAlb was not reduced in peritoneal dialyzed patients in coherence with higher mean blood urea concentrations [100]. An additional study, performed in a limited number of patients, showed that an extended duration of haemodialysis (three times per week, 7 to 8 h per session over one year) was associated with a greater decrease in cAlb concentrations, with a reduction in average blood urea concentrations [101].

#### 4.2.2. Amino Acid Therapies

Free amino acids (AAs) are prone to carbamylation on their α-amino group or on the nucleophilic groups of their sidechains. They have been characterized as strong cyanate scavengers, hampering protein carbamylation. Accordingly, the albumin carbamylation rate was negatively correlated with free AA concentrations in patients with ESRD [20]. In that respect, amino acid therapies aiming to reduce protein carbamylation were investigated. A pilot study reported that haemodialysis patients receiving AA infusion over 8 weeks had a significant decrease in c-Alb without changes in urea [102]. An ongoing clinical trial of amino acid supplementation in patients with ESRD should provide information about clinical outcomes (NCT02472834).

KDIGO guidelines recommend a low protein diet for CKD patients in order to limit renal damages, uremic syndrome and the progression of complications [103]. Noticeably, in very low protein diets (VLPDs), which are composed of 0.3–0.5 g protein/kg/day, essential AAs and keto-analogues are effective at reducing blood urea concentrations [104,105]. Thus, it has been speculated that VLPDs could lower protein carbamylation, and this has been confirmed by a study showing that a VLPD diet administrated to CKD patients decreased serum protein carbamylation in correlation with relative urea reduction [106].

Nutritional therapies seem promising for limiting the carbamylation process, even if additional research is needed to assess their clinical impact.

#### 4.2.3. Other Strategies

Alternative approaches were proposed to avert protein carbamylation. For example, the co-incubation of LDLs with low concentrations of cyanate and three vitamins (ascorbic acid, α-tocopherol and lycopene) led to the impairment of LDL carbamylation [107]. However, higher concentrations of cyanate reduced this phenomenon. Similarly, it was documented that the co-incubation of LDLs with low concentrations of cyanate and nine flavonoids prevented LDL carbamylation [108,109]. However, the scavenging mechanisms underlying this inhibition of protein carbamylation are still unclear, and additional studies remain necessary.

Other compounds were shown to reduce CDP formation through their anti-inflammatory properties [110,111]. For example, aged mice fed with eicosapentaenoic acid, an omega-3 fatty acid, exhibited a reduction of mitochondrial carbamylated proteins in muscle tissues, likely due to MPO pathway interference [111]. Interestingly, a concomitant improvement of mitochondrial functions was observed, suggesting that carbamylation, driven by inflammation, participates in the age-related decline of mitochondrial functions in mice.

Finally, ibuprofen [112], aspirin [113,114] and bendazac [115] have been shown to chemically modify proteins, resulting in impaired interactions with surrounding cyanate and therefore are likely take part in protein carbamylation prevention.

## 5. CDPs: Biomarkers, Predictors of CKD Progression

**Figure 3:**
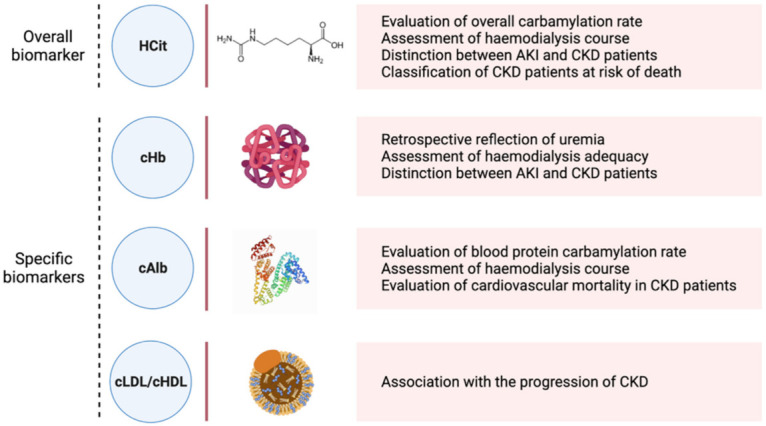
CDPs identified as biomarkers of carbamylation. HCit: homocitrulline; cHb: carbamylated haemoglobin; cAlb: carbamylated albumin; cLDL: carbamylated low-density lipoprotein; cHDL: carbamylated high-density lipoprotein.

As reviewed above, carbamylated proteins participate in the structural and functional changes underlying CKD progression within kidneys, the cardiovascular system, the immune system and the endocrine system. In clinical practice, they have been correlated with the progression degree of the disease and have been identified as promising new biomarkers. The analytical methods used to identify and quantify CDPs have been reviewed elsewhere [19,116]. The biomarkers of carbamylation may be classified in two categories: overall markers, assessed by protein-bound carbamylated lysine (homocitrulline), or specific markers, which correspond to specific carbamylated proteins (Figure 3).

### 5.1. Homocitrulline

The binding of isocyanic acid to the ε-NH2 group of the sidechain of lysine residues generates homocitrulline (HCit), which is used to assess the overall carbamylation rate of serum proteins. The measurement of HCit was demonstrated to have clinical utility in classifying uremic patients at risk of death. Indeed, the plasma levels of protein-bound HCit were higher in haemodialyzed [117] and non-haemodialyzed [118] uremic patients compared to the healthy blood donors [119], and were even greater among patients who died during the 5-year follow-up period [117,118]. Furthermore, HCit was found to be a reliable biomarker in the course of haemodialysis treatment: HCit concentrations decreased by two-fold after 6 months of haemodialysis therapy and were stable during the following 6 months [118]. HCit is also a promising biomarker for distinguishing between AKI and CKD patients, considering that HCit formation depends on urea concentrations and the duration of protein exposure to urea. Indeed, CKD patients exhibited significantly higher HCit values compared to AKI patients in groups with similar blood urea concentrations. Cut-offs were proposed for the CKD/AKI distinction, taking into account the urea concentration to provide better specificity [118].

### 5.2. Carbamylated Haemoglobin

Isocyanic acid binding occurs on the α-NH2 group of the N-terminal valine residues of haemoglobin β chains. As early as the 1980s, carbamylated haemoglobin (cHb) was defined as an informative molecule that could reflect the average concentration of urea in a way analogous to how glycated haemoglobin (HbA1_c_) functions as an integrative retrospective marker of glycemia [120]. Several subsequent studies reported high concentrations of cHb in patients with CKD, with levels decreased in patients on haemodialysis [70,121]. By contrast, patients with AKI exhibit lower cHb concentrations. A cut-off value was proposed for differentiating AKI from CKD [122]. Furthermore, cHb was shown to be relevant to assessing the adequacy of haemodialysis, since cHb was greater in patients with low Kt/V values (≤1.1) compared to those with high Kt/V values (>1.1) [123]. Similarly, there was a negative correlation between cHb and the urea reduction ratio, whereas a positive correlation was observed with the time-averaged urea concentration [38,123,124]. However, cHb concentrations are influenced by anaemia, decreased red blood cell (RBC) lifespan, dialysis-related RBC loss or erythropoietin treatment, which limits the semiological value of this marker [125,126,127].

### 5.3. Carbamylated Albumin

Given that serum albumin is the most abundant protein in plasma, carbamylated albumin was proposed as a means for assessing protein carbamylation extent. Results from a large-scale clinical study revealed a high increase in cAlb in CKD patients compared to non-uremic subjects, with an intense carbamylation of Lys549 residue [20]. Furthermore, cAlb was correlated with cHb and the carbamylation of the unfractionated serum proteins in uremic patients, suggesting that cAlb is a global marker of blood protein carbamylation [20]. The findings also reported that an increase in cAlb was associated with elevated serum markers of cardiac stress, as well as with cardiovascular mortality within 1 and 4 years [20,128]. As cHb, cAlb was found to be an indicator of haemodialysis assessment. Haemodialysis maintenance over 1 year led to a cAlb decrease, which was even greater with an extended haemodialysis [21,101]. Interestingly, cAlb reduction was associated with a reduced left ventricular mass and a survival advantage [21,101]. Finally, the addition of cAlb to the variables differing between patients and controls improved the mortality risk prediction and classification accuracy of patients’ risk [21,88].

### 5.4. Carbamylated Lipoproteins

As mentioned above, the protein component of LDLs, apolipoprotein B, may be targeted by carbamylation. Clinical studies, although performed on small groups, could evidence a 3.3-fold and 2.1-fold increase in cLDLs [28] and cHDLs [45], respectively, in CKD patients compared to healthy subjects, while native lipoproteins concentrations did not vary significantly. Same findings were observed in a larger clinical study in diabetes patients with CKD [61]. There was a progressive increase in plasma carbamylated lipoprotein concentrations as kidney function worsened. Over a period with 9 years of follow-ups, only cHDLs were strongly and independently associated with the progression of kidney disease in type 2 diabetes, suggesting that there is an important contribution of cHDL in the CKD outcomes.

## 6. Perspectives

Despite the investigative efforts made over the past decades, questions remain to be addressed regarding the driving axis and signalling pathways triggered by CDPs. The identification of specific receptors and key intracellular actors would allow for a better understanding of the molecular mechanisms that are induced and a better explanation of the cell behaviours observed in vitro and in vivo. Subsequently, the interception of such pathways with pharmacological strategies would offer an alternative therapeutic approach to hinder CKD progression. Similarly, anti-CDP strategies remain limited, so progress in this field are required. The reduction of CDPs in the body would limit some complications for CKD patients, such as CVD, as discussed above. Important to note is that systemic protein carbamylation was associated with CVD risks in subjects who experienced major adverse cardiac events; however, having preserved kidney functions, the MPO pathway was found to be primary involved [11]. Thus, the reduction of carbamylation induced by hyperuremia, the major source of CDPs, could be an even greater significant benefit for CKD patients. In addition, the use of reliable biomarkers appears critical to assess disease progression and the effectiveness of anti-carbamylation therapies. However, large-scale studies are still needed to confirm the relevance of biomarkers and to provide guidelines for their use in routine practice.

## 7. Conclusions

Even though carbamylation reaction was discovered in the 1960s, it has been intensively investigated only since the 2000s. Nowadays, there is a considerable amount of evidence reporting, first, the adverse effects of carbamylation on protein properties, and second, poor clinical outcomes associated with high CDP concentrations in CKD patients. These data led researchers to consider CDPs as relevant biomarkers associated with the degree of severity of kidney failure. Based on this literature, this review pointed out three major features of carbamylation: (i) the consequence of hyperuremia encountered in CKD patients; (ii) the cause of kidney disease progression through its pathophysiological effects; (iii) a disease tag via the use of CDPs as biomarkers. These three characteristics highlight the significant impact of carbamylation on cellular and molecular mechanisms promoting CKD complications and progression, and the relevance of assaying CDPs in this context. Further investigations are still needed to add insight to the comprehension of molecular mechanisms caused by carbamylated proteins and reinforce the development of therapeutic tools in order to improve clinical outcomes.

## Figures and Tables

**Table 1:** Experimental evidence associating carbamylated proteins and CKD complications.

**CKD** **Complications**	**Carb.** **Compounds**	**Models**		**Key Findings**	**Refs.**
Vascular damages 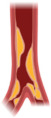	LDL *	Φ	Human leukemic T cells, human fibroblasts	Impairs cLDL binding to the hepatic LDL receptor	[11,39]
		Σ	IV injection in healthy subjects IV injection in rabbits	Delays LDL clearance	
		Φ	Human EPCs, HAECs	Increases EPC senescence Increases HCAEC death via MAPK Uncouples eNOS and reduces NO production Increases ROS production Inhibits angiogenesis	[34,40,41]
		Σ	IV injection in mice	Impairs aortic endothelium-dependent relaxation	
		Φ	HCAECs, human monocyte cell line	Increases expression of ICAM-1 and VCAM-1 on HCAECs Increases monocyte adhesion to endothelial cells via LOX-1	[42,43]
		Φ	HCAECs	Triggers LDL transcytosis via CD-36, SR-A1, SREC-1	[43]
		Σ	IV injection in mice	Induces subendothelial LDL accumulation	
		Φ	Peritoneal macrophages	Promotes lipid loading and foam cell formation through SR-A1	[11]
		Φ	Human CASMCs, human VSMCs	Increases expression of ICAM-1 and VCAM-1 on CASMCs Increases CASMC proliferation Increases VSMC proliferation via SR-A1	[11,42,44]
	HDL *	Φ	Bovine aortic endothelial cells	Induces cell apoptosis	[11]
		Φ	HAECs	Inhibits cell migration and proliferation Inhibits angiogenesis	[45]
		Φ	Human monocyte cell line	Impairs cholesterol efflux Promotes cholesterol accumulation and lipid droplet formation via SR-BI	[46,47]
	Type I collagen	Φ	Human monocytes	Increases monocyte adhesion Increases MMP-9 production and activation	[48]
		Φ	Biochemical assay	Induces local conformational changes in the triple helixImpairs fibrillogenesis	[49]
	Elastin	Σ	ApoE-/- mice fed with cyanate-supplemented water	Increases aortic elastic fibre stiffness Increases aortic pulse wave velocity	[29]
	Mitochondrial proteins *	Φ	Human VSMCs	Promotes mitochondrial dysfunctions and oxidative stress Inhibits ENPP1 and reduces PPi production Increases cell calcification	[26]
		Σ	Nephrectomised rats fed with urea-supplemented diet	Increases aorta calcification by suppressing PPi production	
	Uromodulin	Φ	Human VSMCs	Impairs interaction with and trapping of TNF-α and IL-1β Loses inhibitory effects on osteo-/chondrogenic transdifferentiation	[50]
	Sortilin *	Φ	Human CASMCs	Promotes cell calcification by increasing ALPL and RUNX2 expression and TNAP activity Increases the binding of IL-6, amplifying cell calcification	[51]
		Ψ	Rat aorta	Increases calcification of aortic rings	
Renal Fibrosis 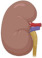	Albumin *	Σ	IP injection in Axolotl	Induces expression of fibronectin and pro-fibrogenic factors (NF-κB, TGF-β1, PDGF-AB, IL-8, ET-1) in tubular cells	[52]
	FBS proteins	Φ	Mesangial cells	Increases cell proliferation Increases synthesis of collagen I and IV	[53]
	Type I Collagen	Φ	Biochemical assay	Increases resistance to MMP-1, MMP-8 and MMP-13	[54]
Haemostasis dysfunctions 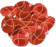	Fibrinogen *	Φ	Biochemical assay	Alters fibrinogen structure Interferes with factor XIIIa-mediated fibrin cross-linking and impairs fibrin polymerization Induces clot resistance to fibrinolysis	[55]
	Fibrino-peptide A	Φ	PMNs	Increases neutrophil chemotaxis	
EPO resistance 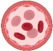	EPO	Φ	Human leukemic cell line	Impairs binding to EPO receptor	[56]
		Σ	SC injections in mice SC injections in rats	Impairs EPO effect on haemoglobin concentrations and haematocrit	[56,57]
Insulin resistance 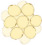	Insulin	Φ	Rat hepatocytes, rat adipocytes	Decreases binding activity Decreases glucose oxidation	[58]
	Free L-Asn *	Φ	Rat adipocytes	Reduces insulin-sensitive glucose uptake	[59]
Immune response disorders 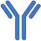	IgG	Φ	Biochemical assay	Impairs C1q binding to IgG Inhibits formation of C4b and C3b	[60]
		Φ	Lymphoma cell line	Decreases cell lysis	
	Type I collagen	Φ	PMNs	Inhibits degranulation and ROS release	[49]

CASMC: coronary artery smooth muscle cells; eNOS: endothelial nitric oxide synthase; EPC: endothelial progenitor cells; EPO: erythropoietin; HAEC: human aortic endothelial cells; HDL: high-density lipoprotein; IP: intraperitoneal; IV: intravenous; LDL: low-density lipoprotein; MMP: matrix metalloproteinase; PMN: polymorphonuclear neutrophils; PPi: pyrophosphate; ROS: reactive oxygen species; SC: subcutaneous; TNAP: tissue nonspecific alkaline phosphatase; VSMC: vascular smooth muscle cells. Φ In vitro studies; Ψ ex vivo studies; Σ in vivo studies; * carbamylated compounds found in patients with CKD [21,26,40,45,51,55,59,61,62].
